# Di­chlorido­dimethyl­bis­(thio­urea-κ*S*)tin(IV)

**DOI:** 10.1107/S1600536814002025

**Published:** 2014-02-08

**Authors:** Yaya Sow, Libasse Diop, Manuel A. Fernandes, Helen Stoeckli-Evans

**Affiliations:** a Laboratoire de Chimie Minerale et Analytique, Departement de Chimie, Faculte des Sciences et Techniques, Universite Cheikh Anta Diop, Dakar, Senegal; b School of Chemistry, Molecular Sciences Institute, University of the Witwatersrand, Private Bag 3, Wits 2050, Johannesburg, South Africa; c Institute of Physics, University of Neuchâtel, Rue Emile-Argand 11, CH-2000 Neuchâtel, Switzerland

## Abstract

The title compound, [Sn(CH_3_)_2_Cl_2_(CH_4_N_2_S)_2_], crystallizes with two half-mol­ecules in the asymmetric unit. Both mol­ecules are completed by inversion symmetry with the two Sn^IV^ atoms located on inversion centers. The metal atoms have distorted octa­hedral coordination environments defined by two Cl atoms, two C atoms of methyl groups and two thio­urea S atoms. In the crystal, mol­ecules are linked *via* N—H⋯Cl and N—H⋯S hydrogen bonds, forming a three-dimensional structure.

## Related literature   

For the applications and biological activity of organotin(IV) compounds, see: Davies (2010 ▶); Evans & Karpel (1984 ▶); Hadjikakou & Hadjiliadis (2009 ▶). For the crystal structures of related compounds, see: Calogero *et al.* (1984 ▶); Sow *et al.* (2012 ▶, 2013 ▶).
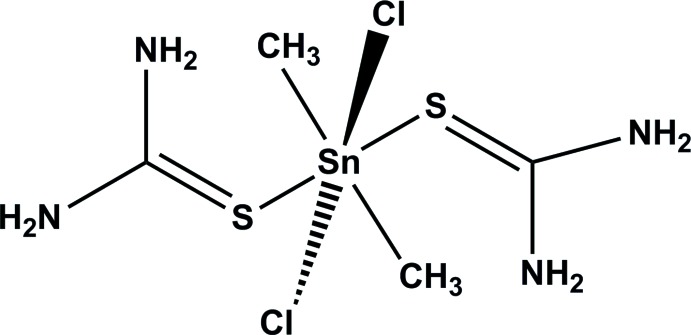


## Experimental   

### 

#### Crystal data  

[Sn(CH_3_)_2_Cl_2_(CH_4_N_2_S)_2_]
*M* *_r_* = 371.90
Triclinic, 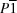

*a* = 6.4461 (1) Å
*b* = 8.4063 (2) Å
*c* = 12.4249 (2) Å
α = 82.172 (1)°
β = 78.240 (1)°
γ = 89.465 (1)°
*V* = 652.89 (2) Å^3^
*Z* = 2
Mo *K*α radiation
μ = 2.65 mm^−1^
*T* = 296 K
0.32 × 0.19 × 0.06 mm

#### Data collection  

Bruker APEXII CCD diffractometer
Absorption correction: integration (by face-indexing with *XPREP*; Bruker, 2005 ▶) *T*_min_ = 0.533, *T*_max_ = 0.827
12611 measured reflections
3250 independent reflections
2917 reflections with *I* > 2σ(*I*)
*R*_int_ = 0.058

#### Refinement  

*R*[*F* ^2^ > 2σ(*F* ^2^)] = 0.021
*wR*(*F* ^2^) = 0.053
*S* = 1.06
3250 reflections
156 parameters
8 restraints
H atoms treated by a mixture of independent and constrained refinement
Δρ_max_ = 0.57 e Å^−3^
Δρ_min_ = −0.54 e Å^−3^

### 

Data collection: *APEX2* (Bruker, 2005 ▶); cell refinement: *SAINT-NT* (Bruker, 2005 ▶); data reduction: *SAINT-NT*; program(s) used to solve structure: *SHELXS97* (Sheldrick, 2008 ▶); program(s) used to refine structure: *SHELXL2013* (Sheldrick, 2008 ▶); molecular graphics: *PLATON* (Spek, 2009 ▶); software used to prepare material for publication: *SHELXL2013* and *PLATON* (Spek, 2009 ▶).

## Supplementary Material

Crystal structure: contains datablock(s) I, global. DOI: 10.1107/S1600536814002025/wm5003sup1.cif

Structure factors: contains datablock(s) I. DOI: 10.1107/S1600536814002025/wm5003Isup2.hkl

CCDC reference:

Additional supporting information:  crystallographic information; 3D view; checkCIF report

## Figures and Tables

**Table 1:** Hydrogen-bond geometry (Å, °)

*D*—H⋯*A*	*D*—H	H⋯*A*	*D*⋯*A*	*D*—H⋯*A*
N1—H1*A*⋯Cl2^i^	0.85 (2)	2.53 (2)	3.3703 (19)	168 (2)
N1—H1*B*⋯Cl2^ii^	0.84 (2)	2.87 (2)	3.4224 (19)	125 (2)
N2—H2*A*⋯Cl2^ii^	0.83 (2)	2.92 (2)	3.5205 (19)	131 (2)
N2—H2*A*⋯S2^ii^	0.83 (2)	2.98 (2)	3.5677 (19)	130 (2)
N2—H2*B*⋯Cl1	0.86 (2)	2.41 (2)	3.255 (2)	170 (2)
N3—H3*A*⋯S1	0.82 (2)	2.54 (2)	3.3522 (19)	173 (2)
N3—H3*B*⋯Cl1^iii^	0.85 (2)	2.60 (2)	3.396 (2)	155 (2)
N4—H4*A*⋯Cl1^iii^	0.84 (2)	2.58 (2)	3.3876 (18)	162 (2)
N4—H4*B*⋯Cl2^iv^	0.86 (2)	2.42 (2)	3.2871 (18)	179 (2)

Symmetry codes: (i) 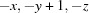; (ii) 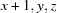; (iii) 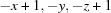; (iv) 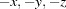.

**Fig. 1.**
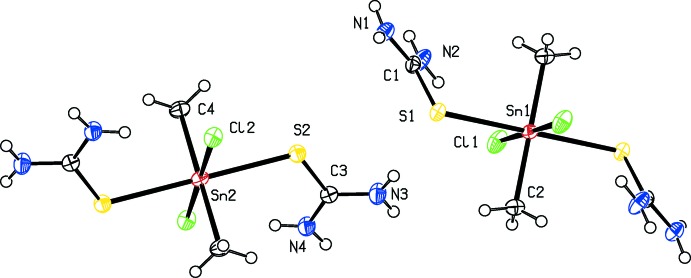
A view of the structures of the two independent molecules of the title compound, with atom labelling. Displacement ellipsoids are drawn at the 50% probability level. Both atoms Sn1 and Sn2 occupy inversion centers. Non-labelled atoms are generated by symmetry codes -x + 1, -y + 1, -z + 1 (Sn1) and -x, -y, -z (Sn2).

**Fig. 2.**
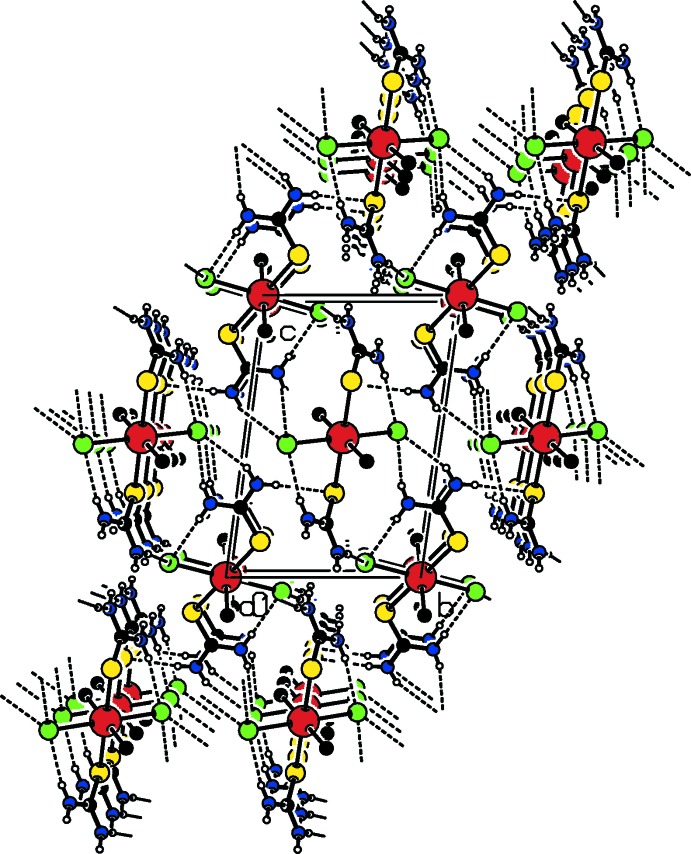
A view along the a axis of the crystal structure of the title compound, showing the crystal packing. Hydrogen bonds are shown as dashed lines (see Table 1 for details).

## supplementary crystallographic information

### 1. Comment

Organotin(IV) compounds have been shown to have several applications in modern technology, for example as catalysis in polymer chemistry (Davies, 2010; Evans & Karpel, 1984). The antiproliferative and anti-tumor activities of organotin(IV) compounds have been reviewed by Hadjikakou & Hadjiliadis (2009). The crystal structures of a number of such compounds involving thiourea ligands have been reported, e.g. Dichloridodimethylbis(1,3-dimethylthiourea-κ*S*)tin(IV) (Calogero *et al.*, 1984), Dichloridodiphenylbis(thiourea-κ*S*)tin(IV) (Sow *et al.*, 2013) and µ_2_-oxalato-bis[triphenyl(thiourea-κ*S*)tin(IV)] (Sow *et al.*, 2012). Herein we report on the synthesis and crystal structure of the title compound, [Sn(CH_3_)_2_Cl_2_(CH_4_N_2_S)_2_].

The molecular structure of the two independent molecules of the title compound is illustrated in Fig. 1. The compound crystallizes with two half molecules in the asymmetric unit. Both molecules possess inversion symmetry with atoms Sn1 and Sn2 located on inversion centers, both with distorted octahedral coordination geometries resulting from two Cl atoms, two C atoms of methyl groups and two thiourea S atoms. The bond lengths and angles are similar to those observed for dichloridodimethylbis(1,3-dimethylthiourea-κ*S*)tin(IV) (Calogero *et al.*, 1984).

In the crystal, molecules are linked *via* N—H···Cl and N—H···S hydrogen bonds into a three-dimensional structure (Table 1 and Fig. 2).

### 2. Experimental

The title compound was synthesized by the reaction of dichloridodimethyltin(IV) with thiourea in a solution of absolute ethanol in a 1:2 molar ratio. After stirring for two hours, a clear solution was obtained. It was allowed to evaporate slowly at room temperature, yielding colourless block-like crystals (yield 63%; m.p. 512 K). Analytical data calculated for C_4_H_14_Cl_2_N_4_S_2_Sn: C 12.92; H: 3.79; N: 15.06; S:17.24; Sn: 31.9 2%: found: C: 12.87; H: 3.63; N: 15.06; S: 16.95; Sn: 31.99%.

### 3. Refinement

The NH_2_ H atoms were located in a difference Fourier map and refined with distance restraints: N—H = 0.86 (2) Å. The C-bound H atoms were included in calculated positions and treated as riding atoms: C—H = 0.96 Å for methyl H atoms, with *U*_iso_(H) = 1.5*U*_eq_(C).

### Crystal data

**Table:**

[Sn(CH_3_)_2_Cl_2_(CH_4_N_2_S)_2_]	*Z* = 2
*M**_r_* = 371.90	*F*(000) = 364
Triclinic, *P*1	*D*_x_ = 1.892 Mg m^−^^3^
Hall symbol: -P 1	Mo *K*α radiation, λ = 0.71073 Å
*a* = 6.4461 (1) Å	Cell parameters from 8182 reflections
*b* = 8.4063 (2) Å	θ = 2.5–28.3°
*c* = 12.4249 (2) Å	µ = 2.65 mm^−^^1^
α = 82.172 (1)°	*T* = 296 K
β = 78.240 (1)°	Block, colourless
γ = 89.465 (1)°	0.32 × 0.19 × 0.06 mm
*V* = 652.89 (2) Å^3^	

### Data collection

**Table:**

Bruker APEXII CCD diffractometer	3250 independent reflections
Radiation source: sealed tube	2917 reflections with *I* > 2σ(*I*)
Graphite monochromator	*R*_int_ = 0.058
φ and ω scans	θ_max_ = 28.3°, θ_min_ = 1.7°
Absorption correction: integration (face-indexed absorption correction carried out with *XPREP*; Bruker, 2005)	*h* = −8→8
*T*_min_ = 0.533, *T*_max_ = 0.827	*k* = −11→11
12611 measured reflections	*l* = −16→16

### Refinement

**Table:**

Refinement on *F*^2^	Secondary atom site location: difference Fourier map
Least-squares matrix: full	Hydrogen site location: mixed
*R*[*F*^2^ > 2σ(*F*^2^)] = 0.021	H atoms treated by a mixture of independent and constrained refinement
*wR*(*F*^2^) = 0.053	*w* = 1/[σ^2^(*F*_o_^2^) + (0.0263*P*)^2^ + 0.0565*P*] where *P* = (*F*_o_^2^ + 2*F*_c_^2^)/3
*S* = 1.06	(Δ/σ)_max_ = 0.001
3250 reflections	Δρ_max_ = 0.57 e Å^−^^3^
156 parameters	Δρ_min_ = −0.54 e Å^−^^3^
8 restraints	Extinction correction: *SHELXL2013* (Sheldrick, 2008), Fc^*^=kFc[1+0.001xFc^2^λ^3^/sin(2θ)]^-1/4^
Primary atom site location: heavy-atom method	Extinction coefficient: 0.0077 (7)

### Special details

**Table:**

Geometry. All e.s.d.'s (except the e.s.d. in the dihedral angle between two l.s. planes) are estimated using the full covariance matrix. The cell e.s.d.'s are taken into account individually in the estimation of e.s.d.'s in distances, angles and torsion angles; correlations between e.s.d.'s in cell parameters are only used when they are defined by crystal symmetry. An approximate (isotropic) treatment of cell e.s.d.'s is used for estimating e.s.d.'s involving l.s. planes.

### Fractional atomic coordinates and isotropic or equivalent isotropic displacement parameters (Å^2^)

**Table:**

	*x*	*y*	*z*	*U*_iso_*/*U*_eq_	
Sn1	0.5000	0.5000	0.5000	0.01799 (7)	
Sn2	0.0000	0.0000	0.0000	0.01831 (7)	
Cl1	0.70413 (9)	0.23120 (6)	0.47747 (4)	0.03146 (13)	
Cl2	−0.11932 (8)	0.29624 (5)	−0.05524 (4)	0.02409 (11)	
S1	0.36555 (8)	0.49581 (6)	0.30643 (4)	0.02033 (11)	
S2	0.19978 (8)	0.15451 (6)	0.13272 (4)	0.02269 (11)	
N1	0.5850 (3)	0.5545 (2)	0.10178 (15)	0.0250 (4)	
H1A	0.473 (3)	0.606 (2)	0.0937 (19)	0.026 (6)*	
H1B	0.691 (3)	0.561 (3)	0.0492 (19)	0.052 (9)*	
N2	0.7433 (3)	0.3848 (2)	0.21896 (16)	0.0300 (4)	
H2A	0.846 (3)	0.385 (3)	0.1668 (17)	0.034 (7)*	
H2B	0.739 (4)	0.333 (3)	0.2837 (15)	0.030 (6)*	
N3	0.2675 (3)	0.0996 (2)	0.33626 (16)	0.0313 (4)	
H3A	0.283 (4)	0.197 (2)	0.334 (2)	0.030 (7)*	
H3B	0.290 (4)	0.040 (3)	0.3937 (16)	0.036 (7)*	
N4	0.1851 (3)	−0.1227 (2)	0.26787 (15)	0.0261 (4)	
H4A	0.198 (4)	−0.169 (3)	0.3298 (15)	0.030 (6)*	
H4B	0.166 (4)	−0.169 (3)	0.2126 (16)	0.026 (6)*	
C1	0.5832 (3)	0.4767 (2)	0.20191 (16)	0.0202 (4)	
C2	0.2200 (3)	0.3664 (3)	0.58135 (18)	0.0294 (5)	
H2D	0.2568	0.2592	0.6075	0.044*	
H2E	0.1275	0.3626	0.5302	0.044*	
H2F	0.1494	0.4172	0.6431	0.044*	
C3	0.2172 (3)	0.0340 (2)	0.25425 (16)	0.0200 (4)	
C4	0.2713 (3)	0.0400 (3)	−0.12940 (17)	0.0261 (4)	
H4D	0.3432	0.1368	−0.1233	0.039*	
H4E	0.2290	0.0503	−0.1997	0.039*	
H4F	0.3648	−0.0489	−0.1239	0.039*	

### Atomic displacement parameters (Å^2^)

**Table:**

	*U*^11^	*U*^22^	*U*^33^	*U*^12^	*U*^13^	*U*^23^
Sn1	0.01928 (10)	0.01734 (10)	0.01622 (10)	−0.00096 (7)	−0.00255 (7)	0.00007 (7)
Sn2	0.01640 (10)	0.01877 (10)	0.01806 (11)	0.00430 (7)	−0.00106 (7)	−0.00052 (7)
Cl1	0.0455 (3)	0.0274 (3)	0.0217 (3)	0.0133 (2)	−0.0094 (2)	−0.0012 (2)
Cl2	0.0289 (3)	0.0200 (2)	0.0240 (3)	0.00809 (19)	−0.0076 (2)	−0.00258 (19)
S1	0.0230 (2)	0.0217 (2)	0.0166 (2)	0.00450 (19)	−0.00449 (18)	−0.00357 (18)
S2	0.0292 (3)	0.0181 (2)	0.0216 (2)	0.00022 (19)	−0.0098 (2)	0.00136 (19)
N1	0.0265 (9)	0.0296 (9)	0.0183 (9)	0.0052 (8)	−0.0047 (7)	−0.0009 (7)
N2	0.0308 (10)	0.0398 (11)	0.0181 (9)	0.0166 (8)	−0.0029 (8)	−0.0037 (8)
N3	0.0539 (13)	0.0191 (9)	0.0235 (10)	−0.0018 (9)	−0.0149 (9)	−0.0005 (8)
N4	0.0379 (10)	0.0195 (9)	0.0221 (10)	−0.0003 (7)	−0.0115 (8)	0.0012 (7)
C1	0.0258 (10)	0.0181 (9)	0.0173 (9)	0.0014 (8)	−0.0052 (7)	−0.0039 (7)
C2	0.0271 (11)	0.0343 (12)	0.0244 (11)	−0.0083 (9)	−0.0016 (9)	−0.0003 (9)
C3	0.0182 (9)	0.0201 (9)	0.0215 (10)	0.0036 (7)	−0.0037 (7)	−0.0020 (8)
C4	0.0232 (10)	0.0272 (10)	0.0247 (11)	0.0024 (8)	0.0014 (8)	−0.0021 (9)

### Geometric parameters (Å, º)

**Table:**

Sn1—C2^i^	2.127 (2)	N1—H1B	0.838 (17)
Sn1—C2	2.127 (2)	N2—C1	1.318 (3)
Sn1—Cl1^i^	2.6234 (5)	N2—H2A	0.828 (16)
Sn1—Cl1	2.6234 (5)	N2—H2B	0.855 (16)
Sn1—S1	2.7221 (5)	N3—C3	1.319 (3)
Sn1—S1^i^	2.7222 (5)	N3—H3A	0.818 (16)
Sn2—C4	2.115 (2)	N3—H3B	0.853 (16)
Sn2—C4^ii^	2.115 (2)	N4—C3	1.318 (3)
Sn2—Cl2^ii^	2.6416 (4)	N4—H4A	0.835 (16)
Sn2—Cl2	2.6416 (4)	N4—H4B	0.863 (15)
Sn2—S2^ii^	2.7468 (5)	C2—H2D	0.9600
Sn2—S2	2.7468 (5)	C2—H2E	0.9600
S1—C1	1.729 (2)	C2—H2F	0.9600
S2—C3	1.723 (2)	C4—H4D	0.9600
N1—C1	1.322 (3)	C4—H4E	0.9600
N1—H1A	0.853 (15)	C4—H4F	0.9600
			
C2^i^—Sn1—C2	180.0	C3—S2—Sn2	112.12 (7)
C2^i^—Sn1—Cl1^i^	89.90 (7)	C1—N1—H1A	116.1 (16)
C2—Sn1—Cl1^i^	90.10 (7)	C1—N1—H1B	124 (2)
C2^i^—Sn1—Cl1	90.10 (7)	H1A—N1—H1B	120 (2)
C2—Sn1—Cl1	89.90 (7)	C1—N2—H2A	117.5 (18)
Cl1^i^—Sn1—Cl1	180.00 (2)	C1—N2—H2B	118.6 (16)
C2^i^—Sn1—S1	92.49 (6)	H2A—N2—H2B	124 (2)
C2—Sn1—S1	87.51 (6)	C3—N3—H3A	123.0 (17)
Cl1^i^—Sn1—S1	88.204 (15)	C3—N3—H3B	119.6 (17)
Cl1—Sn1—S1	91.796 (15)	H3A—N3—H3B	117 (2)
C2^i^—Sn1—S1^i^	87.51 (6)	C3—N4—H4A	114.2 (16)
C2—Sn1—S1^i^	92.49 (6)	C3—N4—H4B	119.5 (16)
Cl1^i^—Sn1—S1^i^	91.795 (15)	H4A—N4—H4B	126 (2)
Cl1—Sn1—S1^i^	88.204 (15)	N2—C1—N1	119.65 (19)
S1—Sn1—S1^i^	180.0	N2—C1—S1	121.92 (15)
C4—Sn2—C4^ii^	180.0	N1—C1—S1	118.41 (15)
C4—Sn2—Cl2^ii^	90.67 (6)	Sn1—C2—H2D	109.5
C4^ii^—Sn2—Cl2^ii^	89.33 (6)	Sn1—C2—H2E	109.5
C4—Sn2—Cl2	89.33 (6)	H2D—C2—H2E	109.5
C4^ii^—Sn2—Cl2	90.67 (6)	Sn1—C2—H2F	109.5
Cl2^ii^—Sn2—Cl2	180.00 (2)	H2D—C2—H2F	109.5
C4—Sn2—S2^ii^	90.20 (6)	H2E—C2—H2F	109.5
C4^ii^—Sn2—S2^ii^	89.80 (6)	N4—C3—N3	118.47 (18)
Cl2^ii^—Sn2—S2^ii^	81.026 (14)	N4—C3—S2	122.29 (15)
Cl2—Sn2—S2^ii^	98.974 (14)	N3—C3—S2	119.23 (15)
C4—Sn2—S2	89.80 (6)	Sn2—C4—H4D	109.5
C4^ii^—Sn2—S2	90.20 (6)	Sn2—C4—H4E	109.5
Cl2^ii^—Sn2—S2	98.975 (14)	H4D—C4—H4E	109.5
Cl2—Sn2—S2	81.025 (14)	Sn2—C4—H4F	109.5
S2^ii^—Sn2—S2	180.0	H4D—C4—H4F	109.5
C1—S1—Sn1	108.77 (6)	H4E—C4—H4F	109.5
			
Sn1—S1—C1—N2	37.99 (18)	Sn2—S2—C3—N4	15.2 (2)
Sn1—S1—C1—N1	−143.46 (14)	Sn2—S2—C3—N3	−165.86 (15)

Symmetry codes: (i) −*x*+1, −*y*+1, −*z*+1; (ii) −*x*, −*y*, −*z*.

### Hydrogen-bond geometry (Å, º)

**Table:**

*D*—H···*A*	*D*—H	H···*A*	*D*···*A*	*D*—H···*A*
N1—H1*A*···Cl2^iii^	0.85 (2)	2.53 (2)	3.3703 (19)	168 (2)
N1—H1*B*···Cl2^iv^	0.84 (2)	2.87 (2)	3.4224 (19)	125 (2)
N2—H2*A*···Cl2^iv^	0.83 (2)	2.92 (2)	3.5205 (19)	131 (2)
N2—H2*A*···S2^iv^	0.83 (2)	2.98 (2)	3.5677 (19)	130 (2)
N2—H2*B*···Cl1	0.86 (2)	2.41 (2)	3.255 (2)	170 (2)
N3—H3*A*···S1	0.82 (2)	2.54 (2)	3.3522 (19)	173 (2)
N3—H3*B*···Cl1^v^	0.85 (2)	2.60 (2)	3.396 (2)	155 (2)
N4—H4*A*···Cl1^v^	0.84 (2)	2.58 (2)	3.3876 (18)	162 (2)
N4—H4*B*···Cl2^ii^	0.86 (2)	2.42 (2)	3.2871 (18)	179 (2)

Symmetry codes: (ii) −*x*, −*y*, −*z*; (iii) −*x*, −*y*+1, −*z*; (iv) *x*+1, *y*, *z*; (v) −*x*+1, −*y*, −*z*+1.
